# Structure-Bioactivity Relationships of Methylxanthines: Trying to Make Sense of All the Promises and the Drawbacks

**DOI:** 10.3390/molecules21080974

**Published:** 2016-07-27

**Authors:** João P. Monteiro, Marco G. Alves, Pedro F. Oliveira, Branca M. Silva

**Affiliations:** 1 Laboratory of Cell Biology and Unit for Multidisciplinary Research in Biomedicine (UMIB), Department of Microscopy, Institute of Biomedical Sciences Abel Salazar (ICBAS), University of Porto, 4050-313 Porto, Portugal; jpspmonteiro@yahoo.com (J.P.M.); pfobox@gmail.com (P.F.O.); 2 Health Sciences Research Centre (CICS–UBI), University of Beira Interior, 6201-506 Covilhã, Portugal; alvesmarc@gmail.com; 3 Institute of Health Research an Innovation, University of Porto, 4200-135 Porto, Portugal

**Keywords:** caffeine, cancer, diabetes, methylxanthine, neurodegenerative diseases, structure-activity relationship, theobromine, theophylline

## Abstract

Methylxanthines are a group of phytochemicals derived from the purine base xanthine and obtained from plant secondary metabolism. They are unobtrusively included in daily diet in common products as coffee, tea, energetic drinks, or chocolate. Caffeine is by far the most studied methylxanthine either in animal or epidemiologic studies. Theophylline and theobromine are other relevant methylxanthines also commonly available in the aforementioned sources. There are many disseminated myths about methylxanthines but there is increased scientific knowledge to discuss all the controversy and promise shown by these intriguing phytochemicals. In fact, many beneficial physiologic outcomes have been suggested for methylxanthines in areas as important and diverse as neurodegenerative and respiratory diseases, diabetes or cancer. However, there have always been toxicity concerns with methylxanthine (over)consumption and pharmacologic applications. Herein, we explore the structure-bioactivity relationships to bring light those enumerated effects. The potential shown by methylxanthines in such a wide range of conditions should substantiate many other scientific endeavors that may highlight their adequacy as adjuvant therapy agents and may contribute to the advent of functional foods. Newly designed targeted molecules based on methylxanthine structure may originate more specific and effective outcomes.

## 1. Introduction

Xanthines are compounds that are produced by both plants and animals. They have not yet been studied as thoroughly as other substances involved in metabolism, despite belonging to the purines family. Xanthine is in fact commonly considered the point of convergence for the purine base metabolism since both adenine and guanine nucleotides converge at this common intermediate [1,2]. Xanthine is involved in the catabolism of nucleotides and nucleic acids, since it is the precursor of uric acid, the final product of the catabolism of the purines [3].

Methylated xanthines (or methylxanthines) are produced in considerable amounts in a limited number of botanical species, including tea (*Camellia sinensis* L.), coffee (*Coffea* sp.) and cacao (*Theobroma cacao* L.), as we will further develop in Section 3 of this review. The most relevant methylxanthines are caffeine, theobromine and theophylline. It has been proposed that plants started biosynthesizing methylxanthines as protection against pathogens and predators, namely insects [4,5].

Methylxanthine consumption is generalized nowadays and started a long time ago. Historical and anthropological data suggest that it may be the result of a human quest for foods/beverages that contained nutrients and/or substances with added value in terms of well-being, other than just calorically [6]. In fact, other than the more common coffee, tea or cacao, other methylxanthine-containing beverages have been used in different cultures, like tejate, which has been used ceremonially in Mexico since pre-Hispanic times [7]. Caffeine and theophylline (this one typically at lower quantities) are present in coffee, tea, cola beverages and chocolate. Theobromine is also present in chocolate products and tea, and is also a caffeine metabolite in humans and other animal species. Paraxanthine, another important caffeine metabolite, is not found naturally in foods [8]. Other than the historically common methylxanthine sources, there are other products that are quickly growing in public preference that deserve careful attention. That is the case of energetic drinks and many food supplements formulated with these ingredients, which are thriving in Western societies right now.

There are a number of widespread myths about methylxanthines. Accepting the stipulation that moderate coffee consumption ranges from 1–3 cups per day (representing roughly 300 mg, if considering instant coffee) and high consumption from 3–6 cups (up to 600 mg/day), one first rather disseminated myth prompts moderate caffeine consumption to be perceived by some to be bordering on dangerous. Therefore, there are those abstaining from drinking coffee and other caffeine-containing drinks based on the assumption that caffeine is (only) bad for you, and that caffeine has no positive effects. This myth will be debunked later in this review. Another common myth would be that decaffeinated coffee does not contain caffeine at all, while in fact it still contains it, although in considerably smaller amounts (normally <5% of the amount found in caffeinated coffee). Another usual misconception would be the public perception of caffeine content in tea, which is normally considered to be high in black tea, lower in green, and next to nothing in white tea, while in fact, caffeine levels may overlap between tea types.

Caffeine is the more thoroughly studied methylxanthine to date, what reflects the prevalence of its consumption in today’s society. Caffeine was first isolated from tea and coffee in the early 1820s [9], and it is probably one of the earliest known examples of functional ingredients sought after by consumers. Caffeine is widely perceived as a central nervous system (CNS) stimulant, and despite the many associated myths its use has been culturally accepted throughout the ages. It acts like a brain cortex stimulant, and is usually sought for by those looking for a general sense of mental energy, with increased awareness and wakefulness, improved clear thinking and attenuated fatigue [10,11]. Caffeine has received a lot of attention, even from sport regulatory institutions. It was reported as enhancing a wide range of exercise activities from those relying on explosive strength [12], to short-term, high-intensity exercise [13,14], to exercise that depends on aerobic activity and endurance [15,16,17], and its use was regulated by the International Olympic Committee (positive controls for more than 12 mg/mL of urine) [18].

As for theophylline, it showed promise as CNS stimulant, although it is mainly used in respiratory disease therapy (namely chronic obstructive pulmonary disease and asthma [19]). It has also been proposed as having applications as a diuretic [20]. Theobromine has showed significant less CNS activity than caffeine and theophylline, possibly because of physicochemical properties that hinder its distribution in the CNS [21].

Herein, we attempt to accomplish a comprehensive compilation of the reported data available regarding the physiological effects of methylxanthines, with special emphasis on caffeine, theophylline and theobromine. We will review the available literature, focusing on the structure-activity relationships that substantiate both beneficial and toxic effects, contextualize where methylxanthine pharmacologic use stands right now, and envision future developments and applications.

## 2. Definition and Chemical Structures

**Figure 1:**
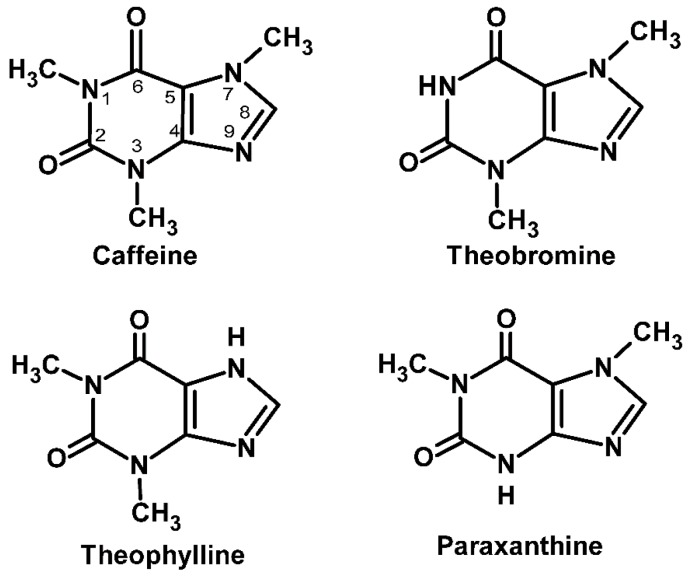
Chemical structures of the three major natural methylxanthines (caffeine, theophylline and theobromine) and paraxanthine.

Xanthine is a purine base found in most human tissues and fluids, as well as in other organisms. Methylxanthines are methylated derivatives of xanthine. They are heterocyclic organic compounds built from coupled pyrimidinedione and imidazole rings [22]. The most relevant naturally occurring methylxanthines (Figure 1) are caffeine (1,3,7-trimethylxanthine), theophylline (1,3-dimethylxanthine) and theobromine (3,7-dimethylxanthine). Paraxanthine (1,7-dimethylxanthine), an isomer of theobromine and theophylline, is not produced by plants but is the major dimethylated byproduct of caffeine. Aminophylline (1,3-dimethyl-7*H*-purine-2,6-dione), pentoxifylline (1-(5-oxohexyl)-3,7-dimethylxanthine), and IBMX (3-isobutyl-1-methylxanthine) are examples of more complexly substituted methylxanthines.

Unlike the others, caffeine was proposed to hold more lipophilic characteristics [21], what should substantiate its ability to easily diffuse through cell membranes and cross the blood–brain barrier [23]. Methylxanthines are weak Brønsted bases and this property should be related with the imino nitrogen at position 9. Theophylline lacks a methyl group at position 7 with regard to the other more relevant methylxanthines, containing instead at that position a proton which can be donated. While caffeine possesses electrophilic sites at positions 1, 3 and 7, theophylline has the same electrophilic predisposition at 1 and 3, in addition to its Brønsted acid site at position 7. The implications of this difference are that, even if both compounds are electron-pair donors, only theophylline is a proton donor in most pharmaceutical systems [24]. Theobromine differs from caffeine by lacking the methyl group at position 1. That single methyl group is enough to confer caffeine different physicochemical properties that were reported to result in substantial increased physiological effects in some contexts [25]. Several synthetic modifications of naturally occurring methylxanthines have been designed with pharmacological intentions, including dyphylline (7-(2,3-dihydroxypropyl)-1,3-dimethyl-3,7-dihydro-1*H*-purine-2,6-dione), proxyphylline (7-(2-hydroxypropyl)-1,3-dimethylpurine-2,6-dione) and enprofylline (3-propyl-7*H*-purine-2,6-dione) [26].

## 3. Natural Sources of Methylxanthines and Biosynthesis

Methylxanthines have been detected in nearly 100 species of 13 orders of the plant kingdom [27,28]. *Coffea* species’ content in caffeine is estimated to be 0.4 to 2.4% dry weight [29]. Caffeine content is 2%–3% dry weight in young leaves of first flush shoots of *Camellia sinensis*, *Camellia assamica* and *Camellia taliensis*, but it represents less than 0.02% in *Camellia kissi* [9]. While in tea (infusion), caffeine may reach between 1.0% and 3.5% of the composition [30,31]. Despite caffeine being generally the major purine present in tea, some *Camellia* species accumulate theobromine instead of caffeine, although cacao (*Theobroma cacao*) is the major natural source of this methylxanthine. Theobromine is, in fact, the predominant one in the seeds of cacao, representing 1.9% [9]. Although considerable amounts of caffeine and theobromine may be consumed from dietary sources (coffee, tea, cola beverages and chocolate) only relatively small amounts of theophylline are thought to be ingested from the same sources [8]. Theophylline occurs naturally in tea and in trace amounts in cocoa and coffee beans [19]. Theobromine levels have been reported to represent only 0.15%–0.46% in different types of chocolate [32]. Young maté (*Ilex paraguariensis* A. St.-Hil.) leaves were reported to contain 0.8% to 0.9% caffeine, 0.08% to 0.16% theobromine and less than 0.02% theophylline. Other plants reported as producing methylxanthines include *Paullinia* sp. (like guarana), *Cola* sp. and *Citrus* sp. [33,34,35,36]. No known plant or food contains paraxanthine [32]. In Table 1, the most common dietary sources and their content in methylxanthines are summarized. Many plants containing methylxanthines, such as coffee, tea, maté, kola nuts, and cocoa beans are commonly used in the production of beverages and foods ingested daily by many people. Tea and coffee are some of the most consumed beverages in the world, and two of the major sources of caffeine intake through diet [37]. Tea is expected to contain less caffeine with regard to coffee [38]. While coffee has been always mostly requested by those seeking a general sense of increased awareness and awakeness, therapeutic connotations have always been suggested for tea. Tea is a complex mixture of about 2000 chemical compounds which include proteins, polysaccharides, minerals and trace elements, organic acids, lignins, polyphenols, methylxanthines and amino acids [39,40]. Within this complex mixture, there are many bioactive compounds believed to promote health benefits [39,41,42,43]. Significant changes were thought to take place in tea composition depending on the type of tea in question and how it has been processed [44]. However, a very recent study by Boros and co-workers reported that caffeine content in commercial teas (white, green, oolong, black, and pu-erh) does not significantly differ according to the processing methods [45].

In plants, methylxanthines are formed from purine nucleotides. Xanthosine, the initial substrate of methylxanthines biosynthesis, may be supplied by different pathways, which include de novo purine biosynthesis (de novo route), degradation of adenine nucleotides (AMP route), the S-adenosyl methionine cycle (SAM route) and guanine nucleotides (GMP route) [55]. Caffeine anabolism is based on steps identical or similar to the anabolism of other methylxanthines. The dominant pathway of caffeine production in higher plants is a xanthosine to 7-methylxanthosine, to 7-methylxanthine, to theobromine, to caffeine pathway [55,56]. This pathway involves consequent methylation of xanthosine, 7-methylxanthine and theobromine and hydrolysis of ribose from 7-methylxanthosine, each onto the next compound of the sequence [55,57]. The enzymes proposed as being involved in the consecutive reactions are 7-methylxanthosine synthase, *N*-methyl nucleosidase, theobromine synthase and caffeine synthase [9,56]. The rate of caffeine biosynthesis is primarily regulated by the induction or repression of *N*-methyltransferases, with special focus on 7-methylxanthosine synthase. Therefore, the rate-limiting step in the caffeine biosynthetic pathway is the initial conversion of xanthosine to 7-methylxanthosine, catalysed by 7-methylxanthosine synthase [55].

In contrast to theobromine, theophylline is more of a catabolite of caffeine than a precursor in plants. Caffeine is slowly degraded by consecutive removal of the three methyl groups, resulting in the formation of xanthine in almost all caffeine-forming plant species, with theophylline being a mid-product of the process [55]. Caffeine [58], theobromine [59] and theophylline [60] may also be obtained by chemical synthesis.

## 4. Extraction, Identification and Quantification

Many different technical approaches have been used to determine the methylxanthines profile of samples of many different types and sources. In fact, it is very easy to find many proposed protocols for the simultaneous determination of the main methylxanthines (caffeine, theobromine and theophylline) in food, beverages and even biological fluids.

Starting with sample preparation, several different pre-treatment protocols have been reported to eliminate unwanted matrix interferences in the determinations, including liquid-liquid extraction [61,62,63,64,65,66], solid-phase extraction [67,68,69,70,71] and even microwaves-assisted extraction [72]. After crude sample pre-treatment, methylxanthine extraction may be achieved by a process including sequential aqueous extraction of raw materials, followed by organic solvent extraction. Water was reported as being a good solvent for methylxanthines, although highly nonselective [73]. Liquid extraction using solvents such as methylene chloride, chloroform, methanol and *n*-hexane, has been used for methylxanthine extraction from natural plants [44,74,75,76,77]. However, most of the more recently pre-treatment methods proposed to be carried out before analytical procedures are rather simpler. In the case of liquid samples (like tea) direct sample application is sometimes an option [50,78,79]. In the case of solid samples (powders, chocolate, leaves), a small amount (in the gram range) is sometimes simply added and extracted in heated (sometimes boiling) stirred water, with posterior filtration [50,71,79,80].

After the pre-treatment and extraction steps, a wide range of analytical techniques have been used for the analysis and quantification of the main methylxanthines including capillary gas chromatography (GC) [81], gas chromatography-mass spectrometry (GC-MS) [82,83], spectrophotometry [77,84,85,86], Fourier transform-Raman spectrometry [87], spectrofluorimetry [88,89], Fourier transform-infrared spectrophotometry [90], capillary electrophoresis (CE) [91,92,93] and micellar electrokinetic electrophoresis (MEKC) [94,95,96], voltammetry [97,98], radioimmunoassay (RIA) [99,100], thin-layer chromatography (TLC) [68,101,102] ion-exchange chromatography [103] and even solid-phase ultraviolet sensing [104]. Supercritical fluid extraction, specifically using supercritical carbon dioxide and ethanol, has been proved to be an effective method for methylxanthine analysis from a number of different samples/sources (guaraná seeds, maté leaves, and cocoa beans) [73]. However, liquid chromatography (LC), more specifically reversed-phase high-performance liquid chromatography (RP-HPLC), has been the most common method of choice used for methylxanthine determination and quantitation. HPLC determination may rely on many different elution modes and mobile phases, as well as detectors. Spectrophotometric detection (DAD or UV, at or about 273 nm, the wavelength normally used to detect xanthines [40,105]) is the most commonly used, but amperometric [106], and mass spectrometric detection [107,108,109] have also been employed. HPLC separation and quantification methods targeted at the simultaneous analysis of the main methylxanthines in samples rely on the use of C18 separation columns and mobile phases with various compositions, including different combinations of several solvents, with the most commonly used mixtures being water + methanol/ethanol + acetic acid [50,78,108] or water + acetonitrile [44,71,109]. Effective separation has been achieved either by isocratic [50,71,78,79,108] or gradient [40,44,109] elution profiles.

More recent technical advances include the use of HPLC methods coupled to mass spectrometry detection. These approaches are very convenient, since they provide structural information and unequivocal identification of the compounds. These mass-spectrometric approaches use positive-mode electrospray ionization (ESI) and may rely on multiple reaction monitoring (MRM) for each of the target methylxanthine species for identification and quantification, monitoring two mass transitions (parent ion and product ion) for each analyte [109]. Several ions [M + H]^+^ have been proposed for identification and quantification purposes [110,111] (Table 2).

## 5. Molecular Targets and Structure-Activity Relationships

All of the naturally occurring methylxanthines have been reported to exert pharmacological effects, the potency of which may be determined by the compound structure itself, the species, the target organ and metabolizing enzyme system idiosyncrasies [32].

Several systematic effects have been described for the main methylxanthines in humans. Caffeine stimulates the CNS and respiratory system, while theophylline is less potent on these targets and theobromine is viewed as virtually inactive in this respect [112]. Theophylline is more effective than caffeine in cardiac stimulation, coronary dilatation and smooth muscle relaxation. As for theobromine, it is generally less active than caffeine or theophylline [112], although it has been reported to be a potent cardiac stimulant, being in fact previously used in humans as a dilator of coronary arteries (daily doses of 300–600 mg) [113]. The relative potencies of these methylxanthines with regard to the aforementioned pharmacological effects is summarized in Table 3. Regarding paraxanthine, its pharmacological effects and toxicological potency on these organ systems were suggested to be negligible [32].

**Figure 2:**
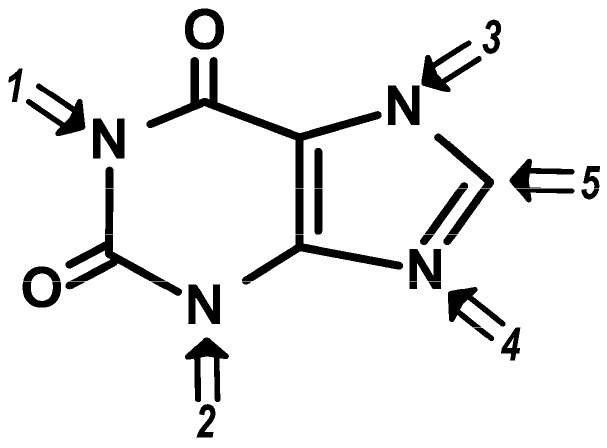
Effects of the structural substitutions of the xanthine molecule on adenosine antagonism and systematic effects: 1. Substitution in position 1 is necessary for high affinity and selectivity towards adenosine receptor sites [116]. 2. Substitution in position 3 increases bronchodilator effect [117,118]. 3. Substitution in position 7 decreases both adenosine receptor antagonism and bronchodilator potency [116,117,119]. 4. Substitution in position 9 results in decreased adenosine receptor affinity [119,120]. 5. Substitution in position 8 increases adenosine antagonism and selectivity towards A_1_ receptors [116,121,122].

Available data helped build a relationship between the potency of each methylxanthine (as presented in Table 3) and the place where substitutions occur in the basic xanthine molecular structure [115] (Figure 2). These evidences imply that strict structure-activity relations modulate the discriminative stimuli elicited by methylxanthines on these specific physiologic effects.

Some of the beneficial effects reported for methylxanthines may be associated with the antioxidant properties ascribed to these compounds. Studies reveal that caffeine is an efficient scavenger of hydroxyl radicals and alkoxyl radicals [123] and this can support the antioxidant role proposed for this compound in protecting against cellular damage by decreasing lipid peroxidation [124,125]. Other than caffeine, theobromine (and xanthine) also exhibit antioxidant properties, and are able to bind and reduce Cu(II) to Cu(I). These properties of caffeine and its metabolites were suggested as contributing to the overall chemopreventive properties of caffeine-containing beverages, such as tea [126].

**Figure 3:**
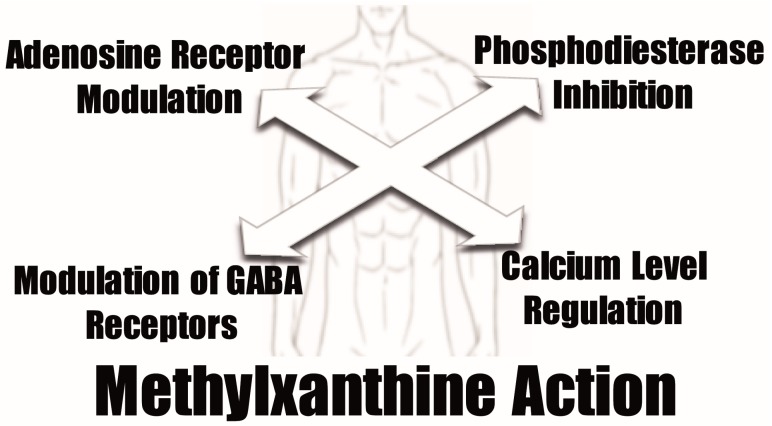
Main mechanisms proposed to mediate the pharmacological activity of methylxanthines at the cellular level.

Despite the positive effects that the antioxidant properties of methylxanthines may originate, their pharmacological activities are normally related to other physiologic activities. Normally, four different mechanisms are proposed to mediate the pharmacological methylxanthine activity at the cellular level: antagonism of adenosine receptors, phosphodiesterase inhibition, modulation of GABA receptor action, and regulation of intracellular calcium levels [127,128,129,130,131,132] (Figure 3).

The most important mechanism of action of methylxanthines involves blocking the adenosine receptors and competitively inhibiting the action of adenosine in the cells. This inhibition results in increased release of hormones, such as norepinephrine, dopamine and serotonin [133]. There are four different types of adenosine receptors and their widespread distribution, fits the proposal of the presence of adenosine in every cell although with differential cell/tissue expression. Adenosine may exert multiple actions in the central nervous, but also on the cardiovascular and even other systems, all depending on the activation of adenosine receptors. Adenosine receptors are classified in terms of their ability to decrease or increase intracellular cAMP concentration. A_1_ and A_3_ receptors are coupled to G_i_ proteins and their stimulation results in decreased intracellular cAMP levels. On the contrary, the stimulation of A_2A_ and A_2B_ receptors increases cAMP levels via Gs proteins [134,135]. Methylxanthines are able to inhibit all four subtypes of adenosine receptors (A_1_, A_2A_, A_2B_ and A_3_), but most of their action is thought to be mediated by inhibition of A_1_ and A_2A_ receptor types (inhibition in the μM range) [11].

The structure-activity relationships for methylxanthines in their antagonism of adenosine have been first studied by Green and Stanberry in the late 70s [136]. These authors demonstrated that the 1-methyl group is pivotal for the inhibitory effects exerted at the adenosine receptor level (increases activity from none to measurable) and interpreted their results on the basis of the antagonism being allosteric rather than competitive in nature. Both caffeine and theophylline are potent inhibitors of adenosine receptors in the human brain. However, theophylline and paraxanthine were proposed to have slightly higher affinities than caffeine for the adenosine A_1_, A_2A_, and A_2B_ receptors [137,138,139,140], and to also be weak antagonists for the adenosine A_3_ receptor subtype [19,141]. While the action of different methylxanthines are not so dissimilar in the case of adenosine receptors of the A_1_ subtype [142], their inhibitory potency is much more diverse in receptors of the A_2_ subtype (A_2A_ plus A_2B_). IC_50_ values for effective blockade of receptors of the A_2_ subtype were 45 and 98 μM for theophylline and caffeine respectively, and 2500 μM for theobromine [143]. Theobromine (not possessing the 1-methyl group reported as important for adenosine antagonism) was in fact reported to have significantly lower affinity than caffeine for A_1_ and A_2A_ receptor subtypes [11,144,145,146].

Despite all the structure-activity variables possible for the interaction between methylxanthines and adenosine receptors, potent and selective antagonists have been developed for all four receptor subtypes based on xanthine structure [147]. Generally, substitutions at position C8 with aryl or cycloalkyl groups has shown promise for identifying novel adenosine A_1_ and A_2A_ receptor antagonists [148]. Other studies concluded that ethyl substitution at the N1, N3 and N7 positions, when compared to methyl substitutions, seemed to enhance adenosine A_1_ receptor affinity [149].

Because for some people methylxanthine ingestion is almost a chronic matter, it is important to note that such exposure may induce effects that resemble the acute effects of adenosine receptor agonists [150], arising from up-regulation of adenosine receptors (A_1_ and A_2A_) and adaptive changes leading to adenosine receptor sensitization [150,151,152].

Methylxanthines may also act as nonselective competitive inhibitors of phosphodiesterases [127,153,154], in particular phosphodiesterase-4 (PDE4) [155,156]. The reversible inhibition of phosphodiesterases compromises the hydrolysis of phosphodiester linkages in different substrate molecules, such as cyclic adenosine cAMP, preventing their degradation and therefore increasing its concentration. In turn, cAMP is an very important second messenger playing fundamental roles in cellular responses to many hormones and neurotransmitters [157].

Several attempts were made to clarify structure-activity relationships of methylxanthines and their potency as phosphodiesterase inhibitors [158]. Caffeine, theobromine and theophylline are all considered relatively weak competitive inhibitors [159]. However, theophylline is supposedly a more potent inhibitor than caffeine [8]. Inhibition of phosphodiesterases was proposed to substantiate the bronchodilator effect of theophylline in asthma treatment [160]. A number of ring-extended xanthines with increased potency (in the nanomolar range) have been developed [161,162]. In fact, structure-activity analyses showed that the methylation at N1 of methylxanthines projects into a small pocket within the phosphodiesterases, and that a pentoxifylline side chain is the largest derivatization fitting that space [163,164].

Regarding the proposed effects of methylxanthines in the modulation of GABA receptors, caffeine [165] and theophylline [166] were both reported to have impact in ion transport by these structures. Later studies specified that caffeine and theophylline act as antagonists or perhaps reverse agonists at benzodiazepine sites, while also interacting with the picrotoxinin and GABA sites [167,168]. A group of xanthine derivatives was studied in order to investigate modulatory effects on the binding of ligands to the benzodiazepine and picrotoxinin sites of GABA_A_ receptors in mouse cerebral cortical membranes, with caffeine having an IC_50_ against [^3^H]-diazepam of 500 μM. Two other xanthine derivatives, 1-propargyl-theobromine and 1,3-dipropargyl-7-methylxanthine revealed to be fivefold more potent than caffeine [167].

Methylxanthines were also proposed to stimulate calcium release from intracellular stores, although at relatively high concentrations, through activation of ryanodine-sensitive calcium channels located in the sarcoplasmic reticulum [128,169,170,171,172]. In fact, caffeine was described as being a full agonist of the ryanodine receptors, forcing Ca^2+^ transient fluxes [167,173,174]. Studies aiming to clarify structure-activity relationships for the efficacy/potency of xanthines in affecting intracellular or intravesicular calcium indicated that disubstituted xanthines (theophylline, paraxanthine) were ineffective (theobromine) or less effective in intracellular calcium elevation than caffeine [171]. Several semisynthetic methylxanthines, including 1-propyl-3,7-dimethylxanthine and 1-propargyl-3,7-dimethylxanthine [171], or 1,3-dimethyl-7-(7-hydroxyoctyl)xanthine and 3-methyl-7-(7-oxooctyl)-1-propargylxanthine [175], were shown to be more potent than caffeine.

From the four mechanisms of action more commonly proposed for methylxanthine physiologic activity, adenosine receptor antagonism should the one with more in vivo relevance [11,159,176]. In fact, methylxanthine plasma concentrations reached through dietary intake should not have effective impact in the activity of phosphodiesterases and GABA_A_ receptors, or calcium release [11,159,177]. At physiological doses (<100 μM) methylxanthines should only be able to act as non-specific adenosine receptor antagonists [11,178], and only at pharmacological doses (in the mM range) would they increase cellular cAMP via inhibition of phosphodiesterases, PDE4 in particular [154,179,180,181]. Only at concentrations exceeding therapeutic levels would methylxanthines interfere with GABA_A_ receptors [11,159]. In summary, methylxanthines are only expected to act as adenosine receptor inhibitors at physiologic concentrations. Other mechanisms of action may also take place, but only in special contexts of methylxanthine supplementation, like the use of methylxanthine-rich supplements or medications [143].

Besides the more commonly proposed mechanisms for the action of methylxanthines, several alternative and/or complementary targets have been more recently disclosed. For instance, inhibitory effects on poly(ADPribose)polymerase-1 (a nuclear enzyme responsible for DNA strand breaks repair) have been proposed, especially by paraxanthine [182]. Also, enzyme assays revealed that methylxanthines are active against human chitinases [183]. Theophylline may activate histone deacetylases at low therapeutic concentrations, especially when their activity is reduced by oxidative stress [184,185], while caffeine may act as a non-competitive inhibitor of acetylcholinesterase [186,187], and has also been reported to be a mild inhibitor of monoamine oxidase B [188].

Interestingly, caffeine, theobromine and theophylline may form non-covalent stacking complexes with ATP [189] and affect DNA and RNA structure [190,191]. The full physiological consequences of these interactions, which should only occur at considerable concentrations anyway, are not clarified yet. Nevertheless, it has been hypothesised that sustained interaction with DNA and RNA after consumption of methylxanthine-containing products may induce or repress gene expression [192].

Methylxanthine activity towards molecular targets is obviously determined by attainable physiological concentrations. Regarding natural methylxanthines pharmacokinetics, they are thought to distribute easily through body fluids, to cross biological membranes and they are metabolized in the liver [193]. Caffeine (and paraxanthine) display shorter half-lives (4.1 and 3.1 h, respectively) than theophylline and theobromine (6.2 and 7.2 h for) as well as higher plasma clearances (2.07 and 2.20 mL·min^−1^·Kg^−1^, for caffeine and paraxanthine respectively, and 0.93 and 1.20 mL·min^−1^·Kg^−1^, for theophylline and theobromine) [194]. Theophylline has a lower volume of distribution at steady state (0.44 L/Kg) when compared to other methylxanthines (0.63–0.72 L/Kg) [194]. After ingestion, caffeine plasma peak concentration was reported to occur at 29.8 ± 8.1 min (after a 5 mg/Kg oral administration) [195]. There are several studies reporting plasma peak concentrations after caffeine ingestion. 10.0 ± 1.0 μg/mL was attained after a 5 mg/Kg oral dose [195], another study reported a concentration of 1.3 μg/mL after consumption of about 80 mg of caffeine (a cup of coffee) [196], and concentrations in the same range were reported after 100 mg caffeine consumption [197,198]. For theophylline, plasma peak concentrations were accessed in therapeutic context and pointed values of 8.4 ± 1.7 mg/L (after oral ingestion of a 5 mg/Kg dose) [199] and of 7 mg/L, following administration of a single dose of 250 mg [200]. As for theobromine, studies are scarcer, if we do not establish a relationship with chocolate consumption. However, peak concentrations between 3.7 to 8.2 mg/L were reported after ingestion of a dose of chocolate containing 240 mg by nursing mothers [201], and a value of 8.05 μg/mL was reported to be attained 2 h after administration dose of chocolate containing 370 mg of theobromine [202].

## 6. Physiological and Health Benefits

In recent years, increased attention has been brought to the dietary effects of methylxanthines. Increased interest from the scientific community, food industry, regular consumers and media on the potential benefits of the consumption of methylxanthine-containing foods and beverages consubstantiated a significant number of basic research and epidemiologic approaches.

The fact that relevant biological effects have been attributed to methylxanthines, supposedly combined with relatively low toxicity, justified the attention dedicated to these compounds and the study of their potential beneficial impact in many disease contexts. In fact, pharmacological formulations containing methylxanthines have been systematically used in common medicine.

Probably, the more obvious target of methylxanthines would be the nervous system. Caffeine has been used as an analgesic co-adjuvant, while being combined with other common analgesics (paracetamol, ibuprofen or acetylsalicylic acid) [203,204]. The anti-inflammatory action of methylxanthines is thought to be related with phosphodiesterase inhibition and/or as a adenosine receptor antagonism mechanisms [205]. However, the most commonly known outcome of methylxanthine consumption (in this case, especially caffeine but not only) would be a psychostimulatory activity [206,207,208]. Moreover, cognitive benefits have also been related with caffeine in both animal and human studies [209,210,211,212,213].

More recently, the study of methylxanthine consumption within the context of neurodegenerative diseases has gathered considerable attention. It is now, undeniably, one of the fields consubstantiating significant methylxanthine research. In fact, regular consumption of caffeine/coffee has been related with lower incidence of Alzheimer’s [214,215,216] and Parkinson’s [217,218,219] diseases. Antagonism of the adenosine receptors (namely A_1_ and/or A_2A_ receptors) was suggested to be the mechanism behind the neuroprotective effects of caffeine [149,220]. However, protection against blood-brain barrier dysfunction may represent a supplementary action justifying such beneficial effects [221,222].

Starting with Alzheimer’s disease, compelling evidence of caffeine neuroprotective activity was compiled in a number of in vitro and in animal studies. In fact, caffeine administration was shown to reduce brain amyloid-β-peptide (Aβ) accumulation in transgenic mice models of Alzheimer’s disease and in cultured neurons taken from these animals [223,224,225,226]. Epidemiologic studies seem to confirm a relationship between regular midlife caffeine/coffee consumption and a decreased risk of developing Alzheimer’s disease [214,216,227,228,229,230]. However, despite all the clues available, the mechanisms underlying caffeine-instigated neuroprotection within the scope of Alzheimer’s disease although very intriguing, remain unfortunately unexplained.

Regarding Parkinson’s disease, both animal [217,231,232] and epidemiologic [218,233,234,235,236,237,238] studies have also hinted a link between midlife coffee/caffeine consumption and lower disease incidence. Anyway, as for Alzheimer’s disease, the actual mechanism by which caffeine-induced neuroprotection is manifested remains unclear. Other than Alzheimer’s and Parkinson’s diseases, caffeine also showed promise in the context of Machado-Joseph disease [239]. Interestingly, it was recently proposed to hold detrimental effects in the scope of Huntington’s disease [240].

Another context in which methylxanthines have been used with therapeutic purposes are respiratory diseases. Curiously, each of the main natural methylxanthines has been used preferentially and with better results in different pathologic conditions. Caffeine is used in the treatment of apnea of prematurity (and its use recently preferred over theophylline) [241,242,243,244,245]. Caffeine action as a ventilatory stimulant has been mostly assigned to antagonism of adenosine receptors in central respiratory centres [246,247,248,249,250,251], although inhibition of cAMP-dependent phosphodiesterase-4 in the neonatal carotid body [252] may also be in play. As for theophylline, it was used since the 1920’s in asthma treatment [19]. Phosphodiesterase inhibition is thought to be a primary mechanism for the bronchodilatory effects ascribed to methylxanthines [19,253], although adenosine receptor antagonism may also be involved in the therapeutic outcomes of methylxanthines asthma treatment [254,255]. Theophylline was also used for some time in the treatment of chronic obstructive pulmonary disease (COPD), although it was lately substituted in this context by other more efficient compounds [256]. Finally, an antitussive action has been unveiled for theobromine [257], which was linked to inhibition of phosphodiesterase activity and antagonism of adenosine A_1_ receptors [257,258].

Although there have always been concerns regarding the effects of methylxanthines (namely caffeine) on cardiovascular parameters, it is now accepted that moderate caffeine consumption does not impact these functions negatively [133,259]. Methylxanthines were reported to hold vasodilator effects [114] and to improve blood microcirculation [260,261]. These effects are supposedly mediated by cAMP levels increase by inhibition of phosphodiesterase activity [259]. Cardiovascular benefits have in fact been ascribed to methylxanthines, supporting their pharmacological use in the treatment of congestive heart failure and anginal syndrome [262]. Moreover, epidemiologic studies have provided evidence of the cardioprotective actions of methylxanthine-containing beverages, in reducing the risk of coronary heart disease and stroke [263,264,265]. Interestingly, theobromine (through chocolate consumption) was shown to increase high-density lipoprotein (HDL) cholesterol, while decreasing plasma low-density lipoprotein (LDL) cholesterol [266,267,268,269,270,271]. This effect is independent of adenosine receptor interference [192] and seems to involve increased levels of apolipoprotein-A-I [271].

Another intriguing field that has motivated interesting methylxanthine research is the field of obesity. Methylxanthines were reported to inhibit the elevation of body fat percentage in the developmental-stage rats [272]. Other reports proposed a lipotropic effect for the three main naturally occurring methylxanthines (caffeine, theophylline, and theobromine) [273], and methylxanthines were in fact described to promote lipolysis in vitro [274,275,276,277]. Moreover, caffeine intake was proposed to be inversely linked to body weight increase in humans [278,279].

Methylxanthines, namely theophylline and caffeine, have been known for a long while to display diuretic and natriuretic effects [280,281]. Adenosine receptor blockade [282,283] and phosphodiesterase inhibition were proposed to be mechanisms involved in these effects [284,285].

Another scope in which methylxanthines have been linked to putative beneficial effects is diabetes. Both coffee [286,287,288,289,290,291,292,293,294] and tea [295,296] regular consumption have been related with decreased type 2 diabetes mellitus incidence. However, caffeine may not be the main bioactive compound present in these beverages that is responsible for such protective effects [294,297,298].

In any case, methylxanthines should be able to actively modulate glucose metabolism [299] since both pancreatic cell insulin secretion [300] and liver glucose output [301] depend on cAMP intracellular levels.

Methylxanthines have also been attributed relevant anti-cancer actions and potential. Caffeine was shown to have the potential to beneficially impact several types of cancer, in a number of studies conducted in animals [302,303,304,305,306,307,308,309] and a number of cancer cell lines [310,311]. Caffeine effects should rely on G0/G1 phase cell cycle arrest in cancer cells [312]. Besides caffeine, also theophylline [313,314,315] and theobromine [155,316,317,318] were reported to hold antitumor potential.

Other than a direct antitumoral effect, methylxanthines also display the ability to act as valid synergistic cancer treatments. In fact, they have been medically used as adjuvant treatments, promoting sensitizing effects when administrated along chemo- [319,320,321,322,323,324,325,326] and radiotherapy [327,328,329] treatments. The synergistic actions of methylxanthines when administrated along other conventional cancer treatments is thought to take place primarily by promotion of arrest or abrogation of the cell cycle checkpoints, namely the G2/M checkpoint [330,331,332,333,334], which results in jeopardized damaged-DNA repair.

Fertility (male fertility in specific) is another sphere in which methylxanthine positive outcomes may be anticipated. Caffeine was shown to improve the nutritional support of spermatogenesis by Sertoli cells [335]. Methylxanthines were also reported to hold beneficial effects in sperm Ca^2+^ transport [336] and in the regulation of cAMP levels, which may correlate with increased motility [337,338]. Methylxanthine containing beverages (namely white tea [339]) and caffeine itself [340], were also reported to be valid additives in the context of sperm storage and in vitro fertilization.

Other than all the aforementioned effects, methylxanthines were also sporadically related with beneficial effects under other contexts. Hepatic (cholestatic liver injury [341] and hepatitis C [342]), kidney (of uric acid nephrolithiasis [343]) and ocular (myopia [344,345]) scopes were also hinted to be positively impacted by methylxanthines, but studies are still scarce and do not justify a proper section for each, at least for now.

## 7. Drawbacks and Toxicity

Despite all the relevant virtues of methylxanthines revealed in the section before, the fact is that some shadows of concern seem to come along with those advantages. Drawbacks, fortunately, seem more circumscribed than promises.

The most pressing concerns about methylxanthine consumption are nowadays related with prenatal exposure. Several studies conducted in animals revealed detrimental methylxanthine actions during pregnancy, related with hindered progeny development [346,347,348]. As for humans, epidemiologic studies comprising analysis of the effects caffeine/coffee consumption in pregnancy parameters, are not at all coherent, some dismissing concerns [349,350,351,352,353], while others do report detrimental effects in development, risk of miscarriage and malformation incidence [354,355,356,357,358]. Concerns seem legitimate, and methylxanthine consumption in pregnant women should be limited and monitored, especially in more aged future mothers [359].

Another aspect that raises concern about methylxanthines regards male fertility, what somewhat contradicts favourable effects presented in the previous section. Animal studies showed that caffeine, theophylline and theobromine administration may induce testicular atrophy and aspermatogenesis [360,361] though the mechanisms remain unknown.

The toxicity of methylxanthines may greatly vary, depending on the specific compound and the animal in question (Table 4). In rats LD_50_s are 200 mg/Kg for caffeine, 206 mg/Kg for theophylline and 950 mg/Kg for theobromine [114]. In humans, the values are relatively close to those in rats with LD_50_’s of 192 mg/Kg for caffeine, and 1000 mg/Kg for theobromine. Human acute toxicity towards methylxanthines is very low. For instance, for caffeine the acute toxic level should be about 10 g/day, which would be comparable to drinking 100 cups of instant coffee [362]. It is important to notice that, in fact, individuals do vary in their sensitivity to methylxanthines, and some of those fluctuations may be genetically originated [363,364,365].

Moderate caffeine consumption is nowadays considered a rather safe habit [366,367]. However, caffeine acute toxicity effects related with excessive intake may occur, and are well characterized. Altered respiratory parameters, gastrointestinal disturbances, insomnia, nervousness, headache, tachycardia, arrhythmia, nausea, seizures [133,368], may be elicited by caffeine intoxication. Caffeine death-related reports are unusual, and imply rather significant concentrations [133,369]. As for chronic effects, they may involve dysfunction of the liver, musculature and the gastrointestinal and renal systems [133,370]. In more extreme cases, symptoms may include myopathy, hypokalemia, muscular weakness, nausea, vomit, diarrhoea and weight loss [207,371]. Another issue related with caffeine chronic consumption as to do with whether it raises dependence or not. Opinions do vary, but a strong argument for those claiming that caffeine does not create dependence is raised by those quoting studies that show that the brain circuitry of dependence are not activated by this methylxanthine [372,373].

Theophylline is normally considered to have stronger toxic effects than caffeine [370]. Theophylline intoxication may manifest in headache, nausea, vomiting, increased acid secretion and gastroesophageal reflux [19], and even convulsions and cardiac arrhythmias at higher concentrations [8,19]. Theobromine has even higher oral lethal doses than caffeine in humans [143], and studies provide evidence of its general innocuousness [374]. However, it may be rather dangerous to other mammals, the most striking example being dogs [258,375]. Since it is commonly accepted that theobromine is generally harmlessness towards humans, studies focusing on its chronic effects are limited. However, we found a study linking undesirable side-effects (sweating, trembling and severe headaches) to long-term intake of considerable quantities of cocoa products (≈100 g cocoa powder per day) [376]. As for paraxanthine it is also supposedly to be fairly harmless for humans [32].

## 8. Conclusions

Methylxanthines have been a more or less noticeable part of human diet for centuries now, and the fact that they are pharmacologically active compounds was disclosed at least about a century ago. Since then, a number of molecular targets have been uncovered, revealing an intricate chemistry for methylxanthines at the cellular level. Such a variety of molecular (and physiological) actions, summarized in Table 5, motivated considerable amount of scientific endeavors that led to far-reaching impact on biomedical research. It seems fair to expect that this trend will continue for a while, since promise shown by these compounds in some therapeutic context is irrefutable, and the fact is that there is still a lot to ponder and clarify.

Anyway, and considering what has been exposed before, it seems sensible to consider that the advantages that methylxanthines may represent in human physiology largely surpass possible detrimental effects. Concerns about possible deleterious outcomes in fertility and prenatal exposure should not be dismissed, although other toxicity concerns should only arise from eccentric intake or maybe pharmacological doses. Idiosyncratic variables should also be taken into account, making predicting methylxanthine toxicity more complex. Detrimental effects that may be idiosyncratically defined include sleep disturbances or interference with the metabolism of certain medications. Nevertheless, moderate methylxanthine consumption, from common sources in diet, should still be considered safe, and maybe even convenient, taking into account all current knowledge.

Some of the existing concerns may be overcome with de novo design of new molecules based on the basic methylxanthine structure, more specific and effective in specific contexts, displaying also lower inherent toxicity. This line of thought has already been followed and a number of methylxanthine-derivatives were (and are) being studied in terms of physiologic potency and toxicity. Hopefully, these structure-activity approaches will prove helpful in the design of new selective and effective drugs targeting specific human conditions. The wide range of methylxanthine molecular targets would make this an appealing field of research. The possibility of architecting/disclosing multiple-target-directed compounds, acting at multiple targets, should also be attractive.

It is expectable that methylxanthines remain valid pharmacological tools, by themselves or as adjuvant treatments, and given the promise reported to methylxanthines in some new disease contexts, other applications may emerge. Moreover, the concept of functional foods is evolving and may as well easily incorporate methylxanthines, in the preparation of foods with putative preventive purposes towards some human conditions. Epidemiologic studies should be expanded, and will help circumscribe relevant target populations for future preventive and therapeutic strategies.

Further technologic developments will provide tools to better understanding methylxanthine physiology, as is now being achieved by neuroimaging techniques, and will make this field of research progress and prosper. Hopefully, the subsequent understanding of methylxanthines molecular and physiologic activities will see them achieve the potential anticipated by current knowledge in some particularly concerning conditions.

## Figures and Tables

**Table 1:** Main sources of methylxanthines in diet.

Dietary Source		*Medium Content (mg)*		
		Caffeine	Theobromine	Theophylline
Coffee	Expresso (23–70 mL)	140 (51–532 range) [46]	-------- *	--------
	Decaffeinated (30 mL)	3.0–15.8 range [47]	--------	--------
Tea	Black (200–235 mL)	55.7 (41.6–71.2 range) [48]	1.8–3.6 [49]	<1 [50]
	Green (200–235 mL)	31.0 (20.2–42.8 range) [48]	0.2–0.7 [51]	--------
Chocolate	Dark (100 g)	0.22–0.31 range [52]	0.26 (0.26–1.16 range) [53]	0–9 [54]
	Milk (100 g)	0.05–0.17 range [52]	0.17 (0.09–0.28 range) [53]	5 [54]
Soft-Drink	Cola (330 mL)	32	--------	--------
	Diet Cola (330 mL)	42	--------	--------
Energy Drinks	Red Bull (250 mL)	80	--------	--------

* Non-detectible or trace amounts. Soft and energy drink caffeine contents were set according to the manufacturers’ specifications.

**Table 2:** Mass spectrometric and MS/MS fragmentation patterns of caffeine, theophylline and theobromine [110].

Methylxanthine	[M + H]^+^	MS Data (MS/MS Fragmentation Pattern)
Caffeine	195.2	181.2, 151.2, 138.0
Theophylline	181.2	167.2, 153.2, 123.5
Theobromine	181.2	167.2, 153.2, 107.5

**Table 3:** Relative pharmacological potencies of the naturally available methylxanthines ranging from more potent (+++) to less potent (+) (adapted from [24,26,114]).

Systematic Effect	Caffeine	Theobromine	Theophylline
CNS Stimulation	+++	+	++
Respiratory Stimulation	+++	+	++
Diuresis	++	+	+++
Coronary Dilatation	+	++	+++
Cardiac Stimulation	+	++	+++
Skeletal Muscle Stimulation	+++	+	++
Smooth Muscle Relaxation (Bronchodilation)	+	+	+++

**Table 4:** Comparative acute toxicity of methylxanthine (adapted from [114]).

LD_50_ (oral, mg/mL)					
	Man	Rat	Mouse	Dog	Cat
Caffeine	150–200 ^a^	200	127	145 ^b^	125 ^b^
Theobromine	1000	950	135 ^b^	300	200
Theophylline	(no data available)	206	332	300	700

^a^ Fatal dose; ^b^ Median lethal dose.

**Table 5:** Methylxanthine reported molecular targets and their suggested involvement in the therapeutic effects ascribed for these compounds.

Molecular Target	Effective Concentrations *	Mediated Therapeutics	Legitimacy of Evidence
Adenosine receptors	50–55 μM for caffeine, 14 μM for theophylline [122]	asthma [254,255], apnea of prematurity [246,247,248,249,250,251], cough [257,258], diuretic [282,283,377], analgesic [204]	from in vitro studies to clinical trials (therapeutic effects)
Phosphodiesterase	55 μM for theophylline [256]	asthma [19,253], chronic obstructive pulmonary disease [256], apnea of prematurity [252], cough [257,258], diuretic [284,285], chronic lymphocytic leucemia [313]	from in vitro studies to clinical trials (therapeutic effects)
GABA receptors	500 μM for caffeine [167]	---------------- **	in vitro studies
Ryanodine-sensitive calcium channels	>1 mM for cafeine [170]	----------------	in vitro studies
Poly(ADPribose) polymerase-1	160 μM for theobromine, 195 μM for theophylline and 200 μM for caffeine [182]	----------------	in vitro studies
Chitinases	469 μM for caffeine and 1.5 mM theophylline [183]	----------------	in vitro studies
Histone deacetylases	24 μM for theophylline [184]	asthma, anti-inflammatory [184]	double-blind crossover controlled study
Acetylcholinesterase	175 μM for cafeine [186]	----------------	in vitro studies
Monoamine oxidase B	700 μM for cafeine [188]	----------------	in vitro studies

* Methylxanthine physiologically-attained concentrations range from 2–50 μM [129]; pharmacological doses should attain plasma concentrations between 55 and 110 μM [256]; ** No reported association with therapeutic effects.

## Abbreviations

The following abbreviations are used in this manuscript:

Aβ	amyloid-β-peptide
AMP	adenosine monophosphate
cAMP	cyclic adenosine monophosphate
CE	capillary electrophoresis
CNS	central nervous system
COPD	chronic obstructive pulmonary disease
GC	gas chromatography
GC-MS	gas chromatography-mass spectrometry
GABA	gamma-aminobutyric acid
GMP	guanosine monophosphate
HDL	high-density lipoprotein
HPLC	high-performance liquid chromatography
LD_50_	median lethal dose
LDL	low-density lipoprotein
MRM	multiple reaction monitoring
MS	mass spectrometry
PDE	phosphodiesterase
SAM	S-adenosyl-l-methionine
TLC	thin-layer chromatography
